# Determination of periodontopathogens in patients with Cri du chat syndrome

**DOI:** 10.4317/medoral.19400

**Published:** 2013-10-13

**Authors:** Sofía Ballesta-Mudarra, Guillermo Machuca-Portillo, Daniel Torres-Lagares, Ángela Rodríguez-Caballero, Rosa M. Yáñez-Vico, Enrique Solano-Reina, Evelio Perea-Pérez

**Affiliations:** 1 Professor. Department of Stomatology, University of Seville, Sevilla, Spain; 2 Assistant Professor of Microbiology. School of Medicine. University of Sevilla; 3 Clinical Assistant in Oral Surgery, University of Seville, Sevilla, Spain; 4 Assistant Professor of Orthodontics and Dentofacial Orthopaedics. School of Dentistry. University of Sevilla; 5 MD. Professor of Microbiology. Chairman of Microbiology. School of Medicine. University of Sevilla

## Abstract

Objectives: Cri du chat syndrome is a genetic alteration associated with some oral pathologies. However, it has not been described previously any clinical relationship between the periodontal disease and the syndrome. The purpose of this comparative study was to compare periodontopathogenic flora in a group with Cri du chat syndrome and another without the síndrome, to assess a potential microbiological predisposition to suffer a periodontitis. Study Design: The study compared nineteen subjects with Cri du chat Syndrome with a control group of nineteen patients without it. All patients were clinically evaluated by periodontal probing, valuing the pocket depth, the clinical attachmente level and bleeding on probing. There were no significant differences between both groups. Aggregatibacter actinomycetemcomitans, Porphyromonas gingivalis, Prevotella intermedia, Tannerella forsythia and Treponema denticola were detected by multiplex-PCR using 16S rDNA (microIDENT). Results: When A. actinomycetemcomitans, P. gingivalis, P. intermedia and T. denticola were compared, no statistically significant differences were found between the two groups (p>0.05). The value of T. forsythia was significantly higher for Cri du chat syndrome (31.6%) than for the control group (5.3%). The odds ratio for T. forsythia was 8.3. Conclusions: In the present study T. forsythia is associated with Cri du chat syndrome subjects and not with healthy subjects.

** Key words:**Cri du Chat syndrome, periodontal health, microbiology, special care dentistry.

## Introduction

Cri du chat syndrome (CdCS)—caused by loss of chromosome 5p-is a genetic alteration associated with oral pathologies (1). The main orofacial abnormalities registered are: mandibular microretrognathia, high—but rarely cleft-palate, variable malocclusion, enamel hypoplasia and retarded tooth eruption (2). All these conditions have been associated with poor oral hygiene. However, there is lack of evident analysing there is periodontal impact of CdCS.

Periodontitis is a chronic infectious disease, affecting almost 40% of the entire adult population (3). Of all the bacteria involved in bacterial biofilms, two species are potentially pathogenic, Prevotella intermedia and Treponema denticola; there are three with particular relevance in the onset and progression of the disease: *Agregatibacter actinomycetemcomitans, Porphyromonas gingivalis and Tannerella forsythia*. These three species cannot be considered the only causative pathogens; rather they are those for which sufficient data is available, and they have been identified in the Consensus Report of the 1996 World Workshop in Periodontics (4). To explain the pathogenesis of periodontitis, attention must be paid not only to the bacteria, but also to the inflammatory responses that they trigger, and to environmental and systemic factors (congenital diseases, systemic syndrome).

Until now, there has been no information about periodontal pathogens in the population with Cri du chat syndrome. Along with the inherent difficulty that this kind of study presents in disabled patients, there is the additional problem of diagnosis. In the past, diagnosis was slow and tedious, mainly due to difficulties in the storage and transportation of samples for an anaerobic culture. A study of a group of these patients-in collaboration with the Spanish Association of Affected Cri du Chat Syndrome Suffers (ASIMAGA)-and the use of molecular techniques that do not require living organisms, have solved these problems. However, it has not been described previously any clinical relationship between the periodontal disease and the syndrome Our purpose was to carry out a comparative study of periodontopathogenic flora in a group with CdCS and one without the síndrome, in order to assess a potential microbiological predisposition to suffer a periodontitis.

## Material and Methods

-Subjects

Subjects with CdCS were compared against a control group. Cri du Chat Group consisted of nineteen patients drawn from the ASIMAGA (7 male and 12 female). There were all of the patients in the association who requested the study.

Control Group consisted of nineteen patients, obtained through the Department of Orthodontics (School of Dentistry, University of Sevilla) without previous orthodontic treatment, including some who were of similar age and sex, were consecutively invited to participate in the study (7 male and 12 female).

Every patient was examined by the same previously trained observer. Number and type of teeth present were recorded. A cali-brated periodontal probe was used to probe four areas of each tooth (mesial, vestibular, distal and lingual). Interproximal areas (mesial and distal) were probed from the buccal aspect to avoid the nauseous or negative reaction which is common in this special kind of patient. The deepest probing depth at each area was recorded.

The present study was carried out with the full knowledge and written consent of each subject (or guardian) and in accordance with the ethical principles governing medical research and human subjects, and with the University of Sevilla Ethical Committee for experimentation approval.

-Detection of pathogenic bacteria.

*Aggregatibacter actinomycetemcomitans* (Aa), *Porphyromonas gingivalis* (Pg), *Prevotella intermedia* (Pi), *Tannerella forsythia* (Tf) and *Treponema denticola* (Td) were detected using PCR. Subgingival samples were obtained by inserting sterile paper points for 2–3 minutes, and were taken in the mesial sulcus of the four first molars.

The samples were pooled and sent to the laboratory within 24 hours of being taken. Upon arrival, the samples were processed; DNA extraction was carried out using the DNeasy Spin Column kit (QIAGEN, Du üsseldorf, Germany). PCR amplification was realized by multiplex PCR using 16S rDNA (microIDENT®, Hain Lifescience, Hehbren, Germany). The cut-off of the test was set, to 103-104 bacteria cells.

-Statistical analysis

Differences between groups were compared using the chi-square test, with statistical significance at p< 0.05. Odds ratios (ORs) were calculated for each variable at p < 0.05. The data obtained were analysed using the SPSS 17.0 software for Windows (LEAD Technologies, Charlotte, NC, USA).

## Results

Periodontal status: no patients were found to suffer from active periodontitis. No sites with a probing depth of >4 mm were found in any of the selected subjects. There were no significant differences between both groups.

The microbiological results obtained are shown in  Table 1. Of all the samples tested, 16 gave positive results for any of the bacteria studied: 8 in the CdCS group and 8 in the control group.

**Table 1:**
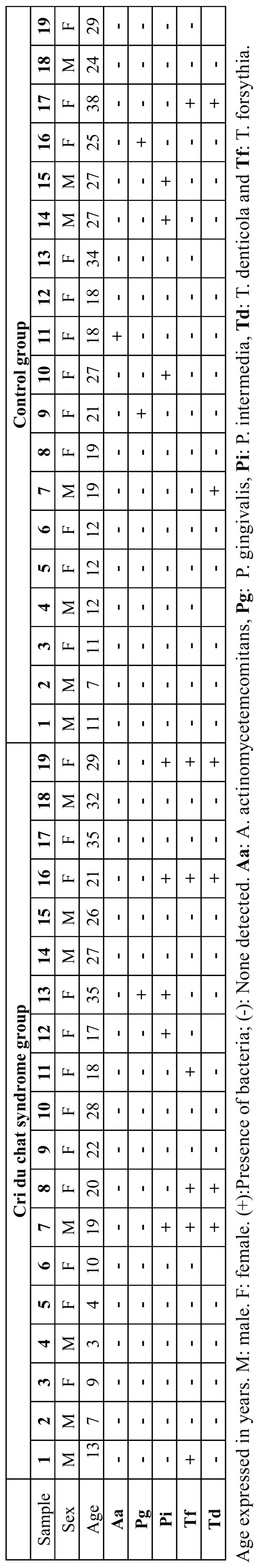
Results of bacteria studied in the Cri du chat syndrome and control groups.

**Figure 1:**
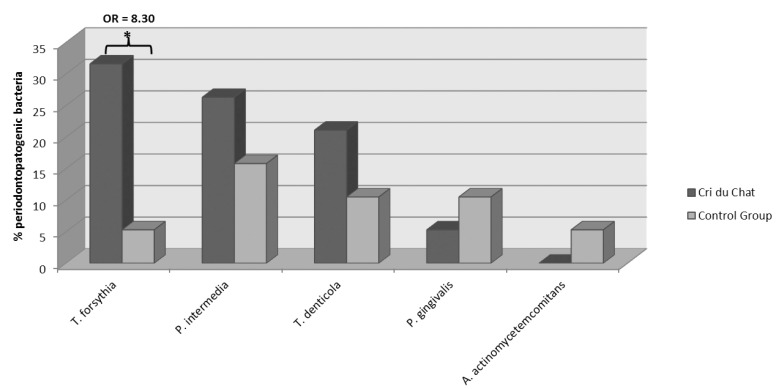
Results obtained for the CdCS and control groups. Data are expressed as percentages of positive sample in the CdCS group, compared to those in the control. * p<0.05 (chi square).

Bacterial pathogen frequencies for the CdCS and control subject groups respectively were: zero and 5.3% for *A. actinomycetemcomitans*; 5.3 and 10.5% for *P. gingivalis*; 26.3 and 15.8% for *P. intermedia*; and 21.1 and 10.5% for *T. denticola* (Fig. 1). There were no statistically significant differences (p>0.05) between the two groups. The value of *T. Forsythia* was significantly higher in the CdCS group (31.6%) than in the control group (5.3%).The odds ratio for *T. forsythia* was 8.3. Data are shown in figure 1.

## Discussion

In 1963, Lejeune et al (5) described a series of common clinical symptoms associated with CdC syndrome. Since then, various authors have studied its clinical and neurological characteristics (6,7), such as a high-pitched cat-like cry, neurological disorders, moon face and microencephaly. A recent study described, for the first time, the craniofacial features including the maxillary and mandibular bones (8) of these subjects, although none have been found regarding oral flora or periodontal status.

As far as our study is concerned, we recognise the limitations due to the small sample size, and the study of some periodonto-pathogens in patients without clinical signs of periodontitis.

Epidemiological studies have estimated CdCS prevalence at 1 in 50,000 live-births, although subsequent studies suggest that it is higher, 1 in 37,000 live births. We know that there are about 350 patients today who live in Spain. Thirty-four patients from different geographical areas of Spain visiting the Department of Dentistry, University of Sevilla (Spain), were invited to participate in this study. Only twenty subjects affected by CdCS met the inclusión criteria.

Together with the inherent difficulties that this kind of study presents regarding patients who are handicapped, there is an additional problem of diagnosis, since more than 700 bacterial species or phylotypes have been detected in the oral cavity, of which more than 50% have not been cultivated (9). The use of molecular techniques which do not require living organisms has resolved this problem.

Several epidemiologic studies (10,11) have examined the relationship between *A. actinomycetemcomitans, P. gingivalis, P. intermedia, T. denticola and T. forsythia* and chronic periodontitis in different populations. Pathogenic bacteria were more prevalent in subjects with chronic periodontitis than in those without periodontitis, although data concerning prevalence is highly variable. Variation between studies could be due to differences in the technique of bacterial detection used, geographic location, ethnicity, age group or periodontal condition of the population samples. In this study, in order to obtain a relatively homogeneous group, the periodontal pathogens of two groups of similar age, sex and periodontal status were compared, and molecular techniques were used.

This is the first known report describing the prevalence of periodontopathogenic bacteria in the CdCS population. When *A. acti-nomycetemcomitans, P. gingivalis, P. intermedia and T. denticola* were compared between the two groups (CdCS and control groups), no statistically significant differences were found. However, the value of *T. forsythia* was significantly higher in the CdCS than in the control group. The microDentA assay has been shown to be highly sensitive and specific for the five test periodontal pathogens (12), However, due the high variability of bacterial genomes, it is possible that certain subtypes might not be detected. Current research (13), which agrees with an earlier report by Socransky (14), indicates that there is a elation-ship—known as “the red complex”- when *P. gingivalis, T denticola and T. forsythia* coexist. However, we found that the probability of being positive for *T. forsythia* was over 8 times greater in the CdCS than in the control group (odds ratio=8.3), although no significant relationship was found between *P. gingivalis, T. denticola* and the CdCS group. Subgingival persistence of *T. forsythia* has also been associated with only a moderate improvement in clinical periodontal status (15). In this study, we excluded subjects with active periodontitis but we cannot rule out past mechanical periodontal debridement treatment. Persistence of *T. forsythia* could be interpreted as a microbial risk factor for a poor treatment response in CdCS patients.

## Conclusion

In summary, these data suggest that *T. forsythia* is closely associated with CdCS subjects, not with healthy ones. Monitoring periodontal microbiology may prove to be a tool in determining the components of microflora involved in the periodontal health of CdCS patients, but today we can´t know the predictive role that could play in patients who do not have clinical symptoms of the disease. Therefore, further research must be performed in order to understand the etiological factors involved in the periodontal status of these patients.
